# Effects of perinatal blood pressure on maternal brain functional connectivity

**DOI:** 10.1371/journal.pone.0203067

**Published:** 2018-08-28

**Authors:** Hiromichi Kurosaki, Katsutoshi Nakahata, Tomohiro Donishi, Michihisa Shiro, Kazuhiko Ino, Masaki Terada, Tomoyuki Kawamata, Yoshiki Kaneoke

**Affiliations:** 1 Department of Anesthesiology, Wakayama Medical University, Wakayama, Japan; 2 Department of System Neurophysiology, Graduate School of Wakayama Medical University, Wakayama, Japan; 3 Department of Obstetrics and Gynecology, Wakayama Medical University, Wakayama, Japan; 4 Wakayama-Minami Radiology Clinic, Wakayama, Japan; University of Texas at Austin, UNITED STATES

## Abstract

Perinatal hypertensive disorder including pre-eclampsia is a systemic syndrome that occurs in 3–5% of pregnant women. It can result in various degrees of brain damage. A recent study suggested that even gestational hypertension without proteinuria can cause cardiovascular or cognitive impairments later in life. We hypothesized that perinatal hypertension affects the brain functional connectivity (FC) regardless of the clinical manifestation of brain functional impairment. In the present study, we analyzed regional global connectivity (rGC) strength (mean cross-correlation coefficient between a brain region and all other regions) using resting-state functional magnetic resonance imaging to clarify brain FC changes associated with perinatal blood pressure using data from 16 women with a normal pregnancy and 21 pregnant women with pre-eclampsia. The rGC values in the bilateral orbitofrontal gyri were negatively correlated with diastolic blood pressure (dBP), which could not be explained by other pre-eclampsia symptoms. The strength of FC seeding at the left orbitofrontal gyrus was negatively correlated with dBP in the anterior cingulate gyri and right middle frontal gyrus. These results suggest that dBP elevation during pregnancy can affect the brain FC. Since FC is known to be associated with various brain functions and diseases, our findings are important for elucidating the neural correlate of cognitive impairments related to hypertension in pregnancy.

## Introduction

Hypertensive disorders of pregnancy are the most common medical complications of pregnancy [1]. Pre-eclampsia is characterized by hypertension and proteinuria; it can result in maternal death, particularly in East Asia [2]. A total of 1/10^th^ of maternal deaths are caused by a hypertensive complication due to pre-eclampsia, which occurs in 3–5% of pregnancies, particularly in patients with nulliparous, diabetes, hypertension, or a chronic disease [3].

Patients with hypertensive disorders, particularly pre-eclampsia, during pregnancy, are reportedly more susceptible to neurological and cerebrovascular symptoms including headache, nausea and vomiting, and visual disturbances [4]. These symptoms are similar to the symptoms of hypertensive encephalopathy [5]. A study focusing on long-term neurocognitive changes in patients with pre-eclampsia showed that women with a history of pre-eclampsia tended to have cognitive impairment later in life [6]. A few neurophysiological studies have focused on central nervous pathophysiology of pre-eclampsia [7]. Although involvement of the frontal subcortical region has been suggested by magnetic resonance imaging (MRI) studies [8, 9], the underlying mechanism has not yet been clarified.

Hypertensive pregnancy disorders not only include pre-eclampsia/eclampsia, but also “only-hypertension status”, which is known as gestational hypertension. Gestational hypertension is characterized by new-onset elevations of blood pressure (BP) after 20 weeks of gestation in the absence of other clinical features, such as proteinuria [1]. Although the outcomes of gestational hypertension are reportedly good in most cases, some women with gestational hypertension experience an elevation in BP to a severe level, with similar outcomes to those observed in women with pre-eclampsia. It has been reported that hypertension in pregnancy increases the future maternal risk of cardiovascular/cognitive disorders [10]. Thus, recent recommendations for the care of pregnant women [1] emphasize the existence of hypertension itself, but not other clinical features (e.g., proteinuria).

Regardless of pregnancy, hypertension is associated with cognitive impairment [11, 12] and white matter lesions [13]. Furthermore, changes in the brain functional network may occur before the onset of a clinical manifestation of cognitive disturbance due to hypertension [14]. In the present study, we hypothesized that hypertension during pregnancy itself could affect brain functional connectivity (FC) regardless of a diagnosis of pre-eclampsia or eclampsia.

To test this hypothesis, we measured regional FC strength using resting-state functional magnetic resonance imaging (fMRI) in women with pre-eclampsia and women with a healthy pregnancy. Analysis of brain FC with fMRI [15, 16] has been shown to be useful in various fields of brain research [17–20]. In the present study, we used voxel-wise whole brain exploratory analysis to localize brain regions in which FC strength is associated with BP. Regional global connectivity (rGC) was measured at each gray matter voxel (6x6x6 mm) that reflects FC strength between a voxel and all other gray matter voxels. Although the rGC is similar to degree centrality, it can be calculated without threshold setting, which could affect FC strength [21–23].

## Methods

### Participants

The current study was approved by the Wakayama Medical University Ethics Committee (No. 1198). Further, all of the participants provided written informed consent. Data from 21 pregnant women with a diagnosis of pre-eclampsia (PE) and 16 healthy control (HC) pregnant women were analyzed. At study entry, all the participants were pregnant and had no history of neurological or psychiatric disorders. All of the participants who enrolled in the present study received routine perinatal care at Wakayama Medical University Hospital between September 2012 and August 2014. Diagnostic criteria for PE were based on international guidelines [2]. The diagnosis included hypertension combined with proteinuria. Hypertension was defined as systolic BP of ≥ 140 mmHg, diastolic BP of ≥ 90 mmHg, or both on two occasions at least 4 hours apart after 20 weeks of gestation. Proteinuria was defined as elevated urinary excretion (i.e., ≥ 300 mg/24 h). In the absence of proteinuria, the diagnosis was established by any of the following: new-onset thrombocytopenia, renal insufficiency, impaired liver function, pulmonary edema, and cerebral and visual symptoms. Severe PE was defined as a systolic BP of ≥160 mmHg or diastolic BP of ≥110 mmHg at least 4 hours apart while the patient was resting on a bed. Gestational hypertension (GH) was defined as new-onset elevations of BP after 20 weeks of gestation in the absence of accompanying proteinuria. Participants with GH were included in the HC group.

**Table 1** outlines the demographic data of the participants. None of the patients had neurological symptoms such as visual disturbance, seizure, or altered mental status. No pathological changes were found by structural MRI in any of the participants. All of the participants with PE were administered antihypertensive drugs including hydralazine hydrochloride, methyldopa, dihydropyridine, and labetalol hydrochloride; parenteral infusion of magnesium, however, was not used. Each drug was prescribed to participants with PE during their hospitalization until the day before delivery. On the day of MRI data acquisition, we confirmed that all of the participants were free from any of the antihypertensive drugs.

**Table 1:** Demographic and clinical characteristics of the participants.

Participant No.	Age (years)	Diagnosis	MRI data acquisition (days after delivery)	Blood pressure (mmHg), systolic/diastolic (mean)	sFlt1/PlGF
1	42	severe PE	5	185/91 (122)	163
2	35	severe PE	6	189/108 (135)	118
3	21	normal	7	135/74 (94)	21
4	34	PE	4	164/101 (122)	300
5	33	severe PE	7	187/115 (139)	1378
6	28	GH	6	152/102 (118)	146
7	34	PE	7	158/92 (114)	44
8	36	Normal	5	115/68 (83)	29
9	29	severe PE	6	168/110 (129)	103
10	30	PE	5	155/89 (111)	294
11	41	normal	3	106/62 (76)	12
12	25	severe PE	5	177/112 (133)	851
13	41	GH	1	147/88 (107)	106
14	36	severe PE	2	187/116 (139)	391
15	32	normal	4	93/53 (66)	37
16	35	normal	5	119/78 (91)	22
17	33	severe PE	7	188/110 (136)	537
18	31	severe PE	5	174/106 (128)	61
19	30	severe PE	3	174/117 (136)	348
20	32	normal	3	114/83 (93)	22
21	29	normal	5	101/76 (84)	3
22	33	normal	3	110/58 (75)	40
23	26	GH	4	188/108 (134)	54
24	29	severe PE	3	163/89 (113)	704
25	41	severe PE	10	177/108 (131)	1859
26	27	severe PE	7	198/118 (144)	1046
27	32	severe PE	4	171/117 (135)	373
28	21	severe PE	9	164/80 (108)	411
29	38	GH	4	148/95 (112)	21
30	39	severe PE	8	198/111 (140)	216
31	29	severe PE	5	173/107 (129)	1158
32	30	normal	3	121/70 (87)	23
33	30	normal	4	107/67 (80)	332
34	30	normal	4	116/90 (98)	57
35	30	normal	6	98/67 (77)	42
36	31	PE	6	165/76 (105)	130
37	35	severe PE	10	203/102 (135)	432

GH, gestational hypertension; PE, pre-eclampsia; PlGF, placental growth factor; sFlt1, soluble fms-like tyrosine kinase-1 receptors

### Blood pressure measurement

Since BP tends to increase until the delivery day and this value is clinically important, we determined the BP of each participant as the highest value on the last day of pregnancy (or delivery date) [24]. All of the participants were inpatients from approximately one week before delivery. Thus, their BP would have been measured in the same conditions. These conditions were as follows: each participant was comfortably seated with legs uncrossed, and the back and arms were supported so that the middle of the cuff on the upper arm was at the level of the right atrium. The participants were instructed to relax and not talk during the measurement.

### Measurement of serum biomarkers

PE-specific serum biomarkers including soluble fms-like tyrosine kinase-1 receptors (sFlt-1) and placental growth factor (PlGF) [25, 26] were measured in all participants. A high ratio of sFlt-1 to PlGF is known to be associated with increased risk of PE. Further, sFlt-1 has been shown to play an essential role in the development of maternal symptoms [27]. Blood samples were collected from the participants by venipuncture in tubes without an anticoagulant immediately after their delivery. Serum was allowed to form, before the samples were centrifuged at 2000 g, pipetted, and stored at -80°C until testing. The concentrations of sFlt-1 and PlGF were measured using an Elecsys 2010 analyzer™ (Roche, Penzberg, Germany) [28].

### MRI data acquisition

An MRI scan was performed within 10 days after delivery. Although MRI for pregnant women is not contraindicated [29],we decided not to perform an MRI scan before delivery because there was no medical merit in performing a head MRI examination for the participants. We assumed that the effect of increase in BP on the brain would last several weeks after delivery, despite decreasing to the normal range at that time.

Brain structural and resting-state functional images of each participant were acquired using a 3 Tesla MRI (PHILIPS, the Netherlands) with a 32-channel head coil (SENSE-Head-32CH). Headphones and earpieces were used to reduce scanner noise. The following parameters were used for T1-weighted structural images: TR = 7 ms, TE = 3.3 ms, FOV = 220 mm, matrix scan = 256, slice thickness = 0.9 mm, and flip angle = 10 degrees. Participants had their eyes closed during the acquisition of all resting-state functional images. A gradient-echo echo-planar pulse sequence sensitive to BOLD contrast [30] was used with the following parameters: TR = 3000 ms, TE = 30 ms, FOV = 192 x 192 mm, matrix scan = 64 x 64, slice thickness = 3 mm, and flip angle = 80 degrees. Three sessions, each with 105 volumes, were performed for each participant. During acquisition, the subjects were instructed to close their eyes and not to move their heads, but not to fall asleep.

### MRI data preprocessing

The functional images were preprocessed using SPM8 software (available at: http://www.fil.ion.ucl.ac.uk/spm) implemented in MATLAB (MathWorks, Inc., Natick, Massachusetts). The first 3 volumes were excluded to allow for T1 equilibration effects, leaving 102 consecutive volumes per session. The slice timing was adjusted to the topmost slice (acquired last) using spline interpolation. To remove gross head motion, rigid body translation and rotation were performed. Further, spatial normalization was achieved using 12-parameter affine transformation to the International Consortium for Brain Mapping Echo-Planar Imaging template in SPM8. The entire session (105 volumes) with either translation of ≥ 2 mm or rotation of ≥ 0.02 radians were not used for further analysis. Each image was resampled to 2-mm isotropic voxels and then smoothed using an 8-mm full-width half-maximum (FWHM) Gaussian kernel.

We used CompCor [31, 32], six head motion time-course parameters regression [33], and global signal regression (mean time course of the functional brain image voxels) to exclude signal artefact unrelated to brain activity [34–36]. Temporal filtering (from 0.01 to 0.1 Hz) was then applied to remove constant offset and linear trends over each run. Structural images were also normalized and resampled to determine voxels of cerebrospinal fluid (CSF), white matter (WM), and gray matter (GM) with a probability threshold of 90%.

### MRI data analysis

Voxels within the GM (down sampled to 6 x 6 x 6 mm) were used to calculate functional connectivity. Functional connectivity between two GM voxels was calculated using Pearson’s coefficient (r) for the 102 functional images. The value of r was then converted to a Z value after effective sample size correction with autocorrelation coefficient values for the voxels [37]. A regional global connectivity (rGC) map was created by calculating each voxel’s weighted degree [21], which was similar to global brain connectivity analysis performed in previous studies [22, 23, 38, 39]. The rGC was computed as the average functional connectivity (Z values) of the voxel with all the other GM voxels. Similar to a previous study [39], we used only positive functional connectivity Z values to calculate the rGC in the current study. This was done because global signal regression could induce artificial negative correlations and low positive (close to zero) functional connectivity could become negative after global signal regression. For each participant, voxel-wise mean rGC values across the three MRI sessions were used for further analyses.

### Correlation between rGC and blood pressure

We first checked the relationship between BP and rGC values to determine the possible effects of BP during pregnancy on brain function. We used the diastolic BP (dBP) for each participant because the value was relatively stable compared to the systolic BP. Further, both the dBP and systolic BP are crucially important for PE diagnosis [1]. Moreover, isolated diastolic hypertension is known to have a stronger relationship than systolic hypertension with systemic complications (e.g., coronary disease), particularly in young women [40]. Treatments for lowering diastolic hypertension are required to decrease systemic complications in young patients.

The BP data distribution was not normal (higher dBP for participants with pre-eclampsia and lower dBP for healthy participants). Thus, a non-parametric permutation test was applied using SnPM13 to assess the correlation between dBP and rGC [41] (SPM toolbox available at: https://www2.warwick.ac.uk/fac/sci/statistics/staff/academic-research/nichols/software/snpm8/). The participant’s age and MRI data acquisition day after delivery were included as nuisance covariates to remove their possible effect. We performed the permutation test 10,000 times to correct for multiple comparisons. Statistical significance was defined as a voxel-wise familywise error (FWE)-corrected p-value < 0.05 (i.e., when the rGC value at the voxel was related to BP).

When we found regions in which rGC values were significantly related to dBP data, functional connectivity (FC) (Z values, see above) between the region (seed) and the rest of the GM voxels was used to specify which regions’ connectivity was related to the dBP data. The seed voxel was defined as the voxel for which the p-value was lowest in the cluster.

Further, we analyzed whether the results could simply be attributed to difference between PE and HC participant groups. First, we checked whether the rGC/FC values of regions found to be significantly related to dBP using all of the subjects’ data were also related dBP values for the HC group. Then, Spearman’s method was used to evaluate the relationship between rGC/FC values and BP in each region for the HC and PE groups. Second, a whole brain analysis was performed using sFlt1/PlGF to check whether rGC was related to PE severity. The relationship between dBP and sFlt1/PlGF values was also assessed using Spearman’s method. We used a natural logarithm of sFlt1/PlGF according to a previous study [42].

### Effects of dBP on vascular coupling of the BOLD signal

The BOLD signal is significantly affected by vascular components such as pulsation and the vascular bed [43]. Thus, a dBP-related FC change could be simply due to the effect of vascular coupling of the BOLD signal. To explore this possibility, we first examined the correlations between (1) dBP and the variance of the global signal and (2) dBP and the variance of the mean white matter signal since both values are substantially related to vascular components [43]. Furthermore, when we found a voxel whose rGC was associated with dBP, we assessed the relationships between the rGC values and the variances of the global signal and mean white matter signal. We also assessed the relationship between rGC and dBP using a partial correlation analysis with Spearman’s method to remove the effects of variances of the global and mean white matter signals.

## Results

**Fig 1:**
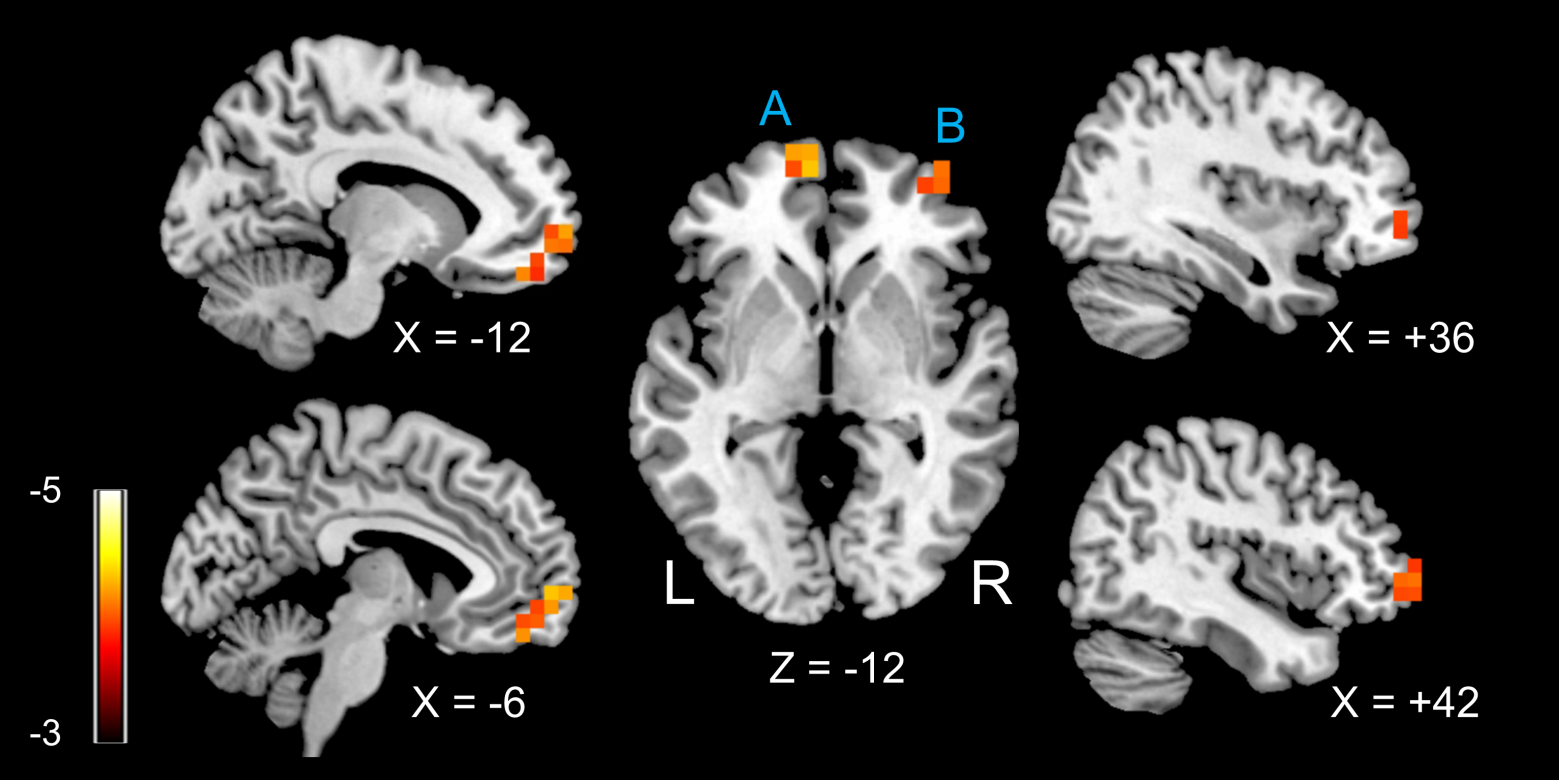
Regions in which regional global connectivity (rGC) values were significantly related to diastolic blood pressure (dBP). Two clusters with significant t-values (FWE-corrected p < 0.05) are shown. A: left medial orbitofrontal area (BA10); B: right middle orbitofrontal area (BA 46). Scale: t-value.

**Fig 2:**
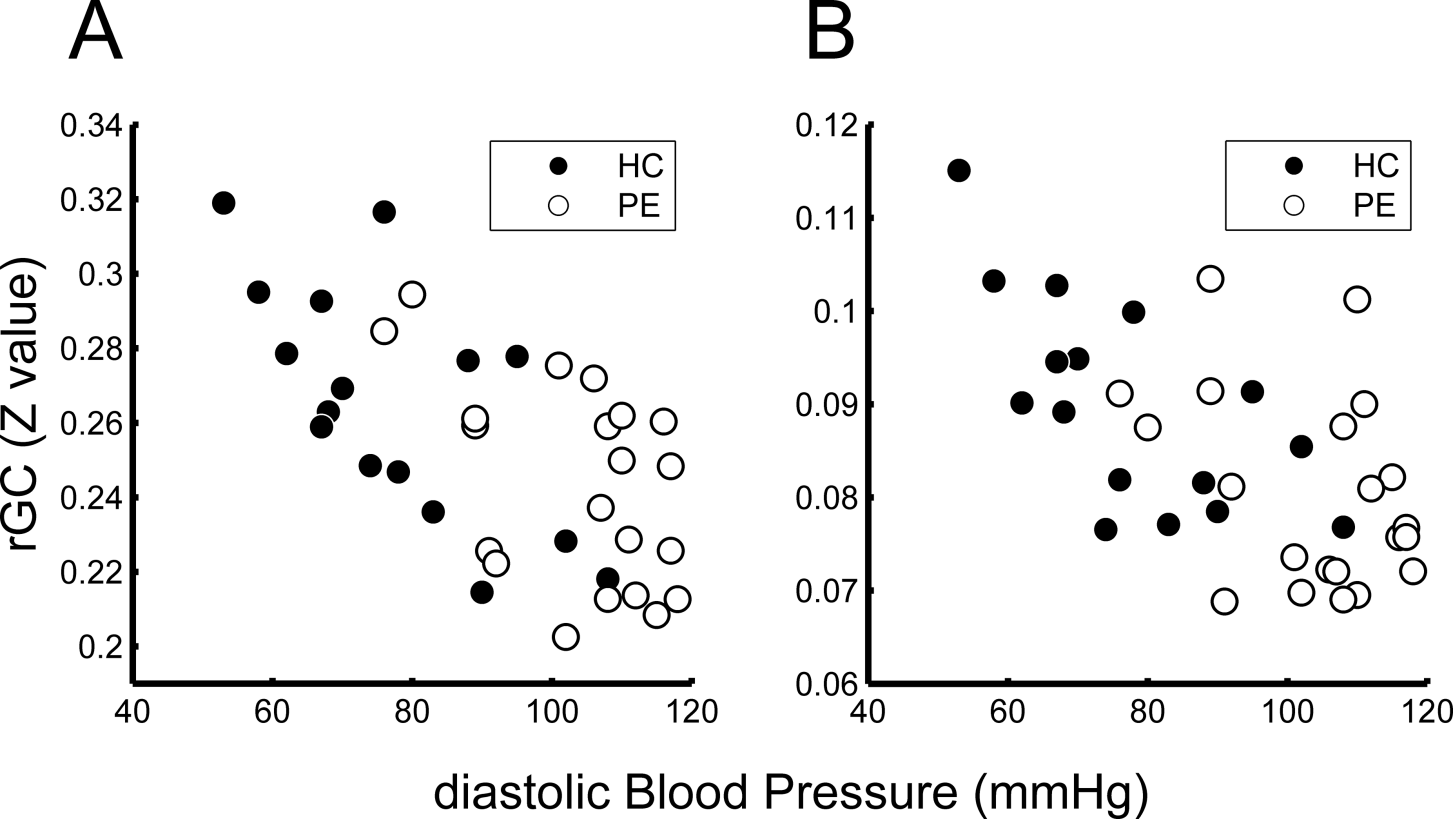
Relationship between diastolic blood pressure (dBP) and regional global connectivity (rGC). Relationship between dBP and rGC values for each participant at the peak voxel of each cluster is shown (A: left medial orbitofrontal area; B: right middle orbitofrontal area). The rGC values were inversely related to dBP. Note that the data for the healthy control (HC) group show the same tendency for both areas (p<0.05 by Spearman’s method, see text).

We found two regions in the frontal lobes (left orbitofrontal gyrus and right orbitofrontal gyrus) in which rGC values were negatively related to dBP for each participant (**Fig 1**). **Table 2** shows the Montreal Neurological Institute (MNI) coordinates and FWE-corrected p-values. **Fig 2** shows the relationship between dBP and the rGC value for each participant at the peak of the two clusters (ROI_A and _B in **Table 2**). For ROI A, the rGC values showed significant negative relationships with dBP as revealed by Spearman’s method, for both the HC and PE participant groups (ROI A: rho = -0.69, p = 0.003 and rho = -0.47, p = 0.03 for the HC and PE groups, respectively). For ROI_B, only the data of the HC group showed a significant negative relationship between rGC and dBP (rho = -0.66, p = 0.005 and rho = -0.22, p = 0.32 for the HC and PE groups, respectively).

**Table 2:** Brain regions in which regional global connectivity (rGC) values were negatively related to diastolic blood pressure (dBP).

ROI	Brain region	Peak MNI coordinates (mm)	Number of voxels	T value	P value	
					uncorrected	FWE-corrected
A	Left medial orbitofrontal gyrus	(-6, 60, 0)	15	-4.50	0.0001	0.0223
B	Right middle orbitofrontal gyrus	(42, 60, 0)	7	-4.31	0.0002	0.0351

MNI, Montreal Neurological institute; ROI, region of interest

**Fig 3:**
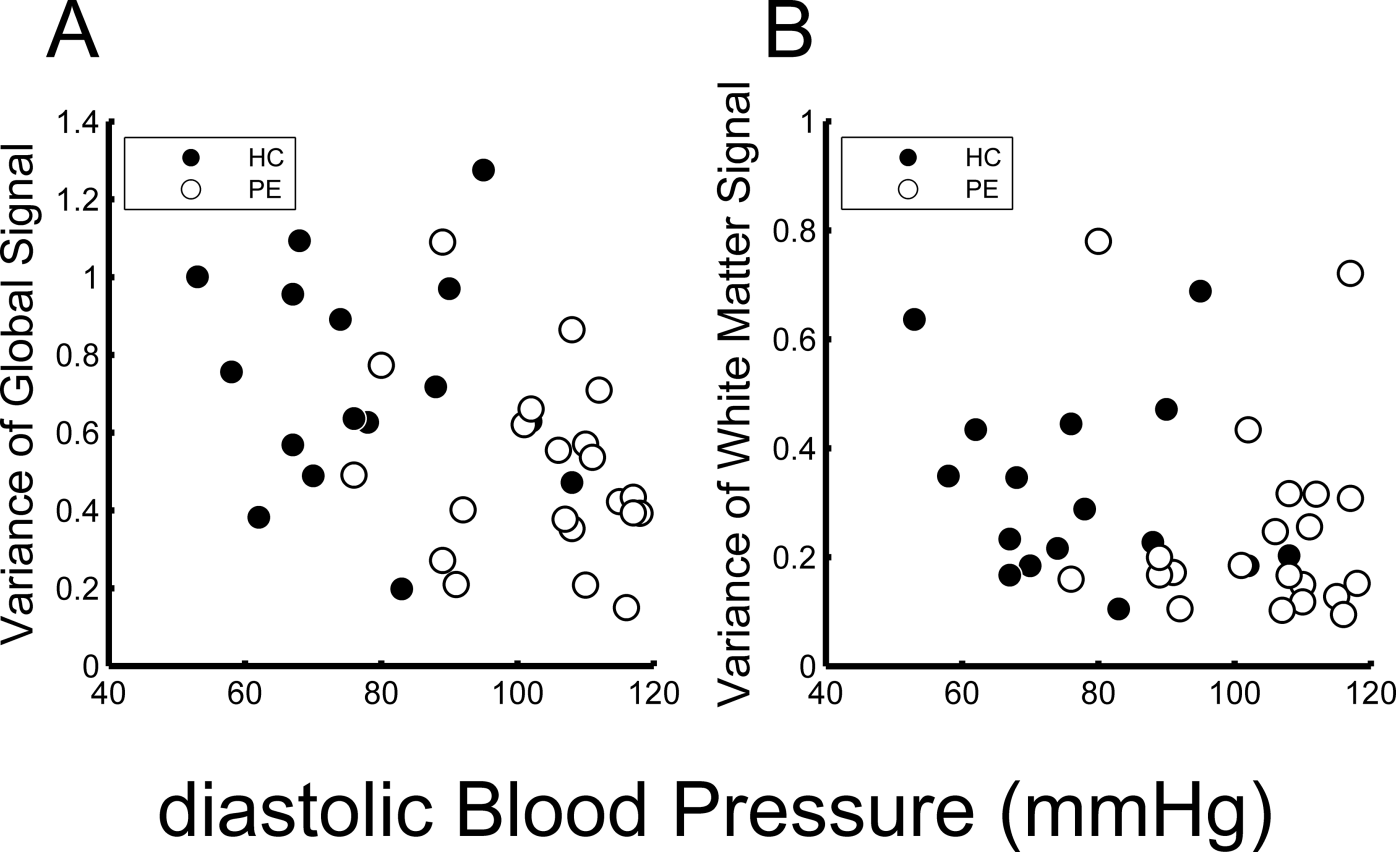
Relationships of diastolic blood pressure (dBP) with global and mean white matter signal variances. Both variances of the global signal (A) and mean white matter signal (B) were inversely related to dBP (A: rho = -0.42, p = 0.0095; B: rho = -0.28, p = 0.09 by Spearman’s method).

**Fig 3** outlines the relationships between dBP and variance of the global signal (**Fig 3A**) and between dBP and variance of the mean white matter signal (**Fig 3B**). The variance of the global signal was significantly inversely related to dBP (**Fig 3A**; rho = -0.42, p = 0.0095 by Spearman’s method). Although variance of the mean white matter signal also tended to decrease with dBP, however, it was not statistically significant (**Fig 3B**; rho = -0.28, p = 0.092 by Spearman’s method).

**Fig 4:**
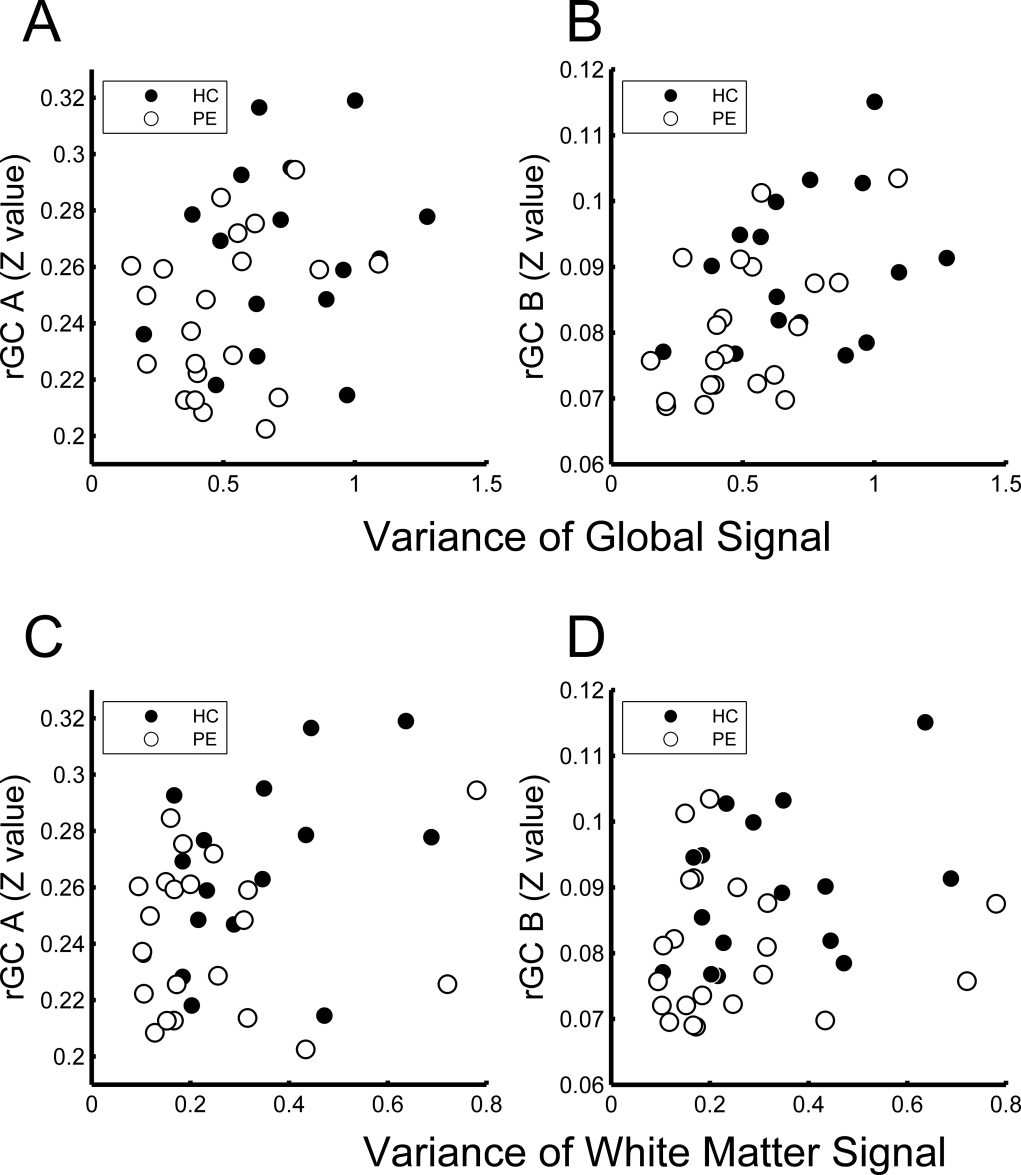
Relationships between variances of global and mean white matter signals and regional global connectivity (rGC) values. Variance of the global signal was related to the value of rGC at ROI_A (rGC A) (rho = 0.34, p = 0.038, by Spearman’s method) (A) and the value of rGC at ROI_B (rGC B) (rho = 0.51, p = 0.0014) (B). Variance of the mean white matter signal was not related to the value of rGC at ROI_A (rho = 0.29, p = 0.09) (C) or ROI_B (rho = 0.27, p = 0.11) (D).

According to the Spearman’s analysis, there were significant positive correlations between the variance of the global signal and rGC for ROI_A (**Fig 4A**; rho = 0.34, p = 0.04) and at ROI_B (**Fig 4B**; rho = 0.51, p = 0.0014). **Fig 4C and Fig 4D** show the relationships between the variance of the mean white matter signal and rGC values of the ROI_A and ROI_B, respectively. According to the Spearman’s analysis, these associations were not significant (rho = 0.29, p = 0.085 for ROI_A and rho = 0.27, p = 0.11 for ROI_B).

Since the rGC values (in ROI_A and ROI_B) increased with elevations in both the variances of global and mean white matter signals, we analyzed the partial correlation between rGC values and dBP, excluding the effects of variances of global and mean white matter signals. The results were essentially the same as the results outlined above. The rGC values were significantly inversely related with dBP (rho = -0.55, p = 0.000675 for ROI_A and rho = -0.46, p = 0.006 for ROI_B). When the data were divided into two groups (HC and PE groups), the rGC values in the HC group were significantly inversely related to dBP (rho = -0.71, p = 0.0047 for ROI_A and rho = -0.69, p = 0.0065 for ROI_B). However, the rGC values of the PE group were not significantly related to dBP (rho = -0.41, p = 0.08 for ROI_A and rho = -0.015, p = 0.95 for ROI_B).

**Fig 5:**
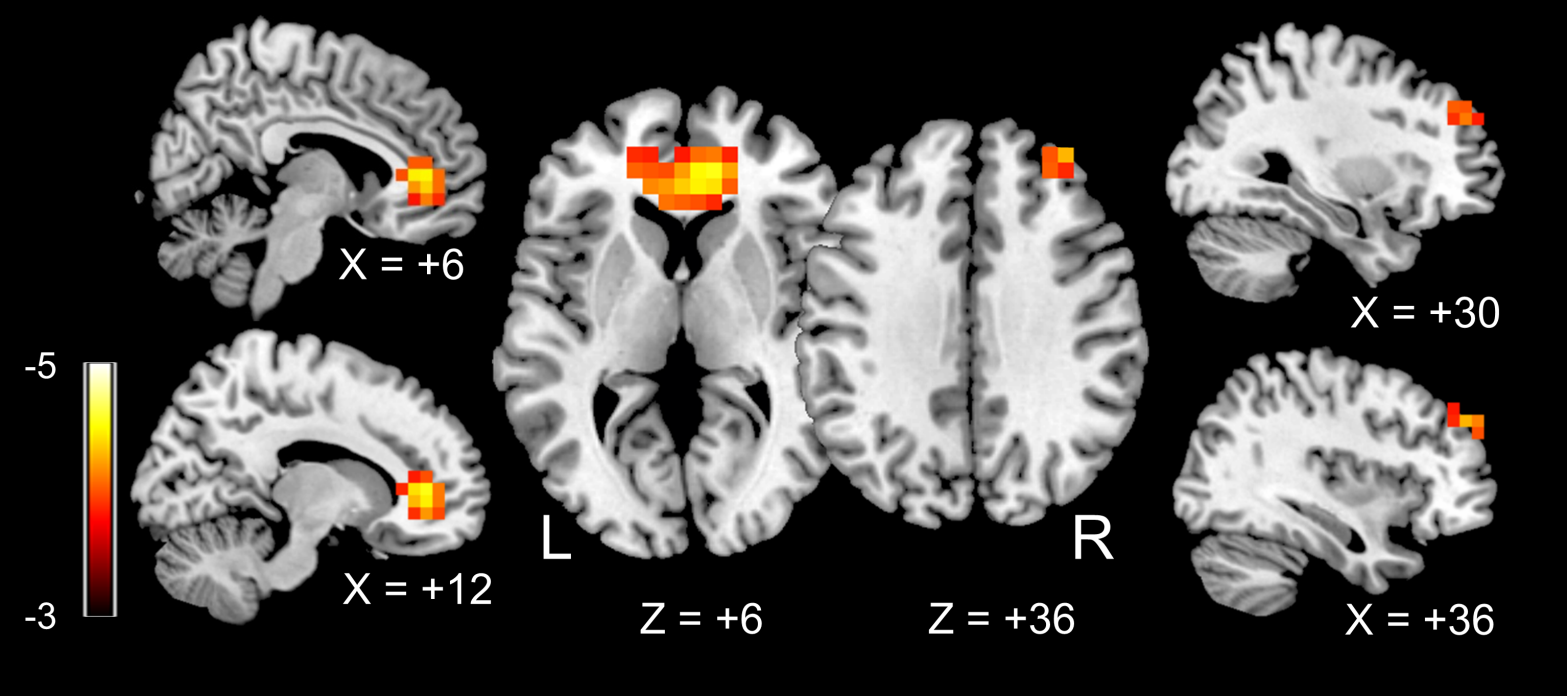
Regions in which functional connectivity (FC) to the left medial orbitofrontal area was related to diastolic blood pressure (dBP). Functional connectivity (FC) values for all of the brain voxels seeding at ROI_A (see Fig 1) were calculated. SnPM revealed that two clusters were significantly related to dBP (FWE-corrected p<0.05): the left and right anterior cingulum gyri and right middle frontal gyrus. Scale: t-value.

We then performed a seed-based FC analysis using the two voxels in ROI_A and ROI_B (see **Table 2**). When the ROI_A peak voxel was used as a seed, we found two regions in the frontal lobes in which the FC values to the seed showed a significant negative relationships with the dBP. These regions were the bilateral anterior cingulate gyri and right middle frontal gyrus (**Fig 5**). **Table 3** outlines the MNI coordinates of the peak voxels and FWE-corrected p-values. However, we did not find any region in which the FC value to the seed ROI_B was related to the dBP.

**Table 3:** Brain regions in which FC values to ROI A were negatively related to dBP.

Brain region	Peak MNI coordinates (mm)	Number of voxels	T value	P value	
				uncorrected	FWE-corrected
Right anterior cingulate gyrus	(12, 42, 6)	62	-5.42	0.0001	0.0061
Right middle frontal gyrus	(36, 48, 36)	10	-5.10	0.0001	0.0136

**Fig 6:**
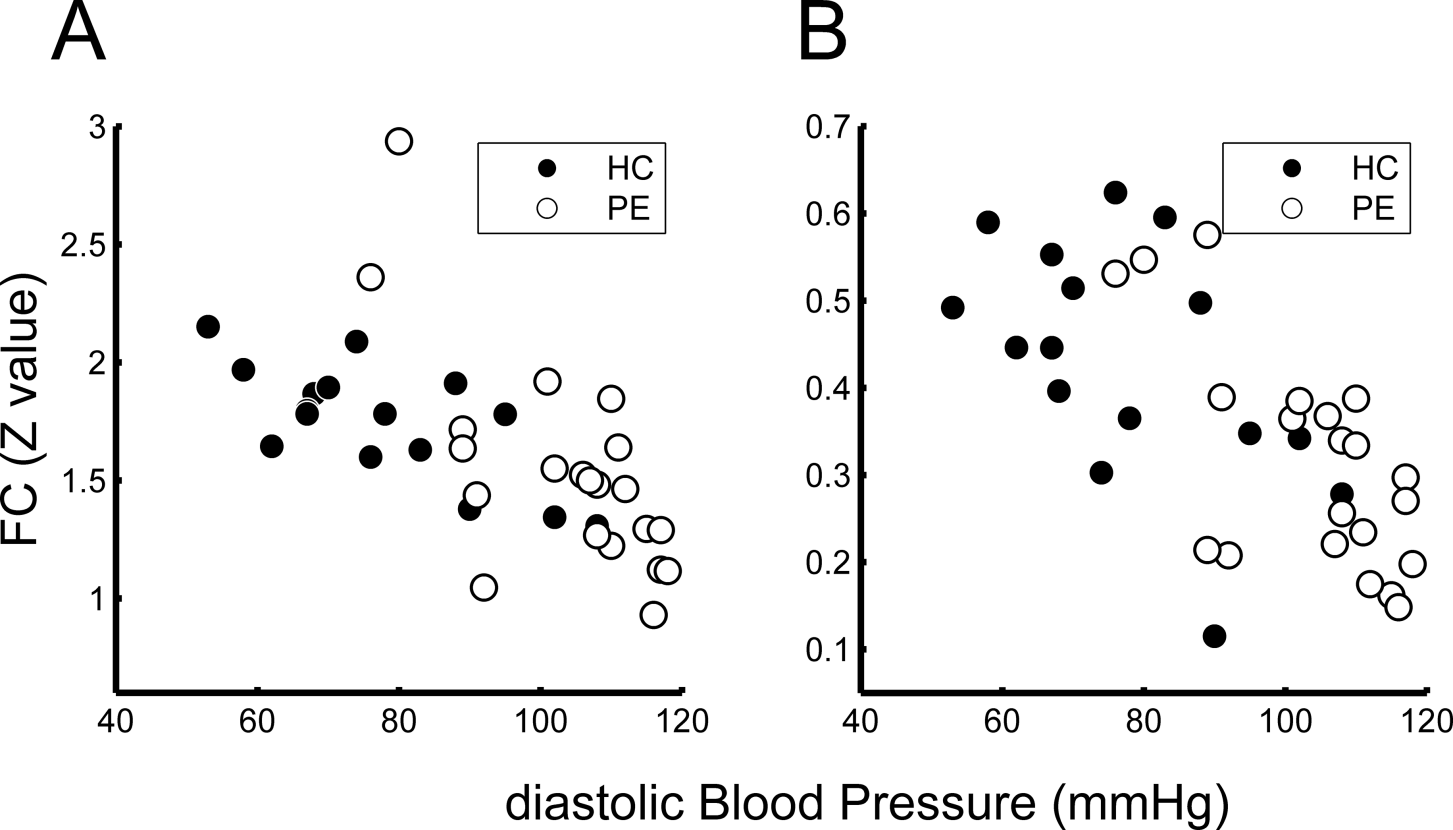
Relationship between functional connectivity (FC) and diastolic blood pressure (dBP) at the peak voxels. FC values from the seed ROI_A (see Fig 1) at the peak voxels of the two clusters (A: right anterior cingulum; B: right middle frontal gyrus, see Fig 5) are shown with dBP for each of the participants. Data for both the healthy control (HC) and pre-eclampsia (PE) groups showed that FC values are related to dBP.

**Fig 6** shows the relationship between FC strength (Z-value, see methods) seeding at ROI_A and the dBP in the two regions. In both regions, Spearman’s method revealed a significant negative relationship between the FC and the dBP, in both the HC and PE participant groups (anterior cingulum: rho = -0.66, p = 0.006 and rho = -0.64, p = 0.002 for the HC and PE groups, respectively, middle frontal: rho = -0.50, p = 0.048 and rho = -0.62, p = 0.003 for the HC and PE groups, respectively).

**Fig 7:**
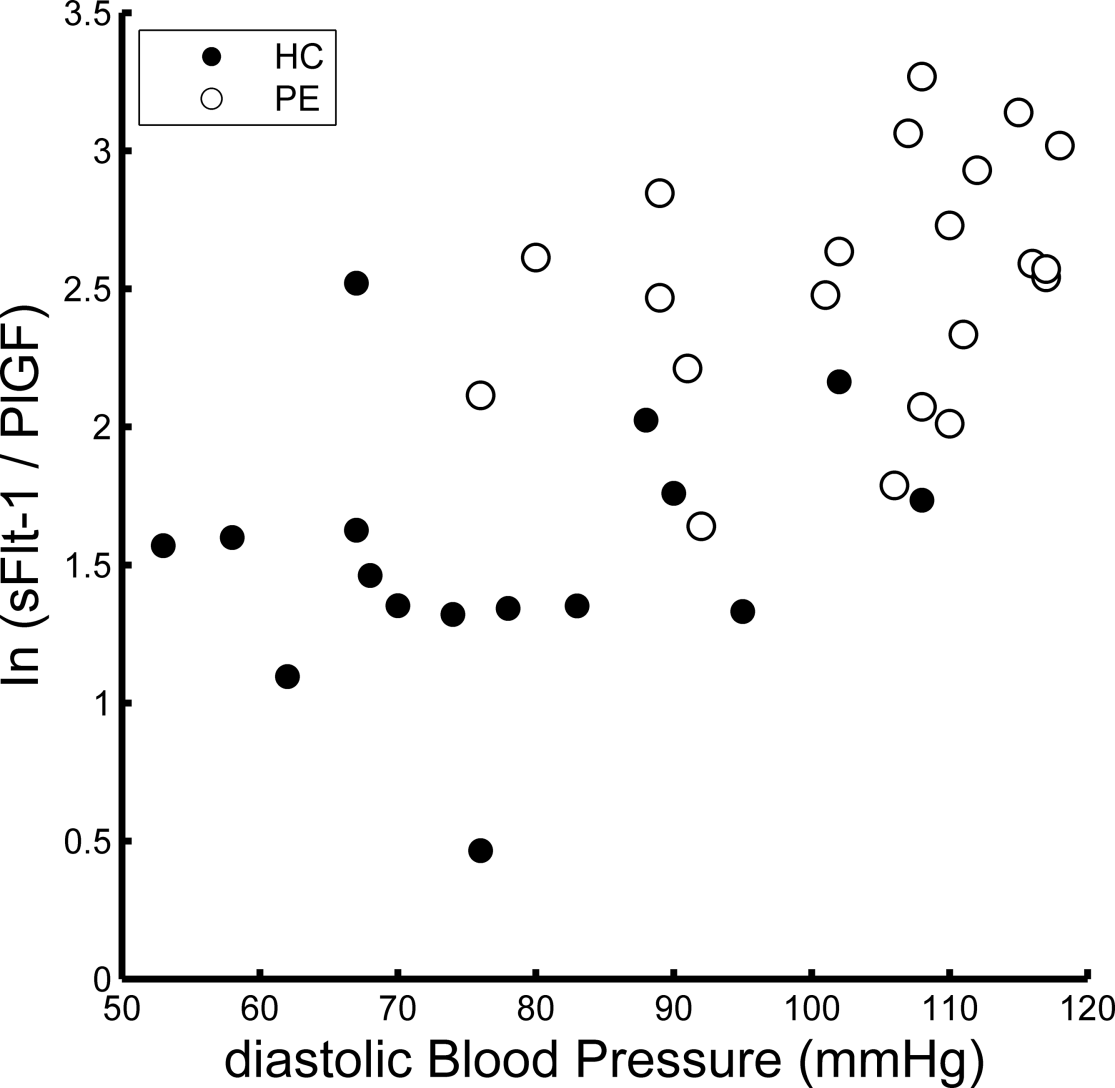
Relationship between diastolic blood pressure (dBP) and sFlt1/PlGF. This figure shows the relationship between dBP and the natural logarithm of sFlt1/PlGF. There was a strong positive correlation between these values (rho = 0.65, p < 0.001, by Spearman’s method).

**Fig 7** shows the relationship between the dBP and sFlt1/PlGF natural logarithm. There was a strong positive correlation between these values (rho = 0.65, p < 0.001, using Spearman’s method). However, there were no brain regions that showed a significant relationship between the rGC and sFlt1/PlGF natural logarithm.

## Discussion

The main findings of the current study are as follows: 1) there was a significant negative correlation between the rGC and dBP in the left medial orbitofrontal gyrus (ROI_A) and right middle orbitofrontal gyrus (ROI_B); 2) the FC of the bilateral anterior cingulate gyri and right middle frontal gyrus seeding at ROI A was negatively related to dBP.

These results cannot be solely attributed to differences in BP between the HC and PE groups. This is because the rGC values of ROI_A and ROI_B were also negatively related to the dBP for each of the HC participants (**Fig 2**). Furthermore, there were no brain regions in which the rGC value was associated with the serum biomarker of PE severity (sFlt-1/PIGF), even though the value was significantly related to dBP (**Fig 7**). These results suggested that a relatively high dBP, even in the HC participants, could affect the brain FC.

It is reasonable to assume that the results were simply due to the effects of vascular coupling of the BOLD signal [43] because the effects would increase with BP elevations. In fact, we did find that the rGC values tended to increase with variances of the global and mean white matter signals. In contrast, we unexpectedly found that both variances decreased with increases in dBP (**Fig 3**). Thus, global and mean white matter signal variances cannot explain the relationship between the rGC and dBP values. This was confirmed by the results of partial correlation analysis, which excluded the effects of global and mean white matter signal variances.

Since global and white matter signals are significantly affected by vascular coupling [43], it is natural to assume that both variances are related to dBP. The discrepancy in our results, however, may indicate that the vascular tone [44] of PE participants was higher than that for HC participants. This in turn would have lowered vascular coupling of the BOLD signal in PE participants compared with that in HC participants. Cerebrovascular resistance increases with elevations in cerebral perfusion pressure (which is related to BP) to maintain cerebral perfusion as observed in pre-eclampsia [45]. Vascular coupling is increased when cerebral perfusion is markedly impaired [46]. Among patients with severe pre-eclampsia, severely increases in blood pressure is known to cause loss of cerebral autoregulation [47]. In such a pathological status, vascular coupling of the BOLD signal would increase. In the participants with PE, the autoregulation mechanism would be maintained, or even have undergone overregulation.

The occipital lobe is thought to be a key structure involved in hypertensive encephalopathy during pregnancy, such as posterior reversible encephalopathy syndrome (PRES). Typically, in these patients, T2-weighted MRI shows a uniform and characteristic pattern in the white and gray matter of the parietal and occipital lobes [48]. The pathogenesis of PRES is thought to be severe hypertension; impaired cerebrovascular autoregulation is likely to be involved [49, 50]. A previous study, which aimed to determine brain areas most vulnerable to a hypertensive state in patients with eclampsia, showed that all MRI-positive patients had possible brain edema in the occipital and parietal regions [51]. Thus, although our participants did not have an MRI abnormality, it is rather surprising that we did not find any parieto-posterior regions in which rGC values were related to dBP. However, a recent study revealed that women who experienced PE had MRI lesions 6–12 months after delivery and these lesions were mainly found in the frontal lobes [8]. Thus, PRES-related brain edema may not be related to white matter lesions [9]. Our findings may indicate that the participants with relatively high dBP had frontal lobe white matter impairments that caused reduced FC (i.e., reduced rGC in the present study) in the ventral frontal regions. In line with that possibility, it was reported that reduced FC associated with long-term treated hypertension was mainly found in areas in the so-called border zone supplied by the middle and anterior cerebral arteries, such as in the cingulate gyrus, ventral frontal areas, and prefrontal areas [52]. Our results also showed reduced FC between the medial orbitofrontal gyrus and anterior cingulate gyrus/middle frontal gyrus (**Fig 5**).

The orbitofrontal cortex is connected to the ventral part of the anterior insula, which constitutes the viscero-autonomic system included in the salience network [53]. Women with a history of PE tend to show cognitive impairment and executive dysfunction [6]. The lower rGC value in the ventral frontal regions could be a neural correlate of the abnormal psychological aspect of emotion perception in women with PE. However, it is not clear whether hypertension in normal pregnancy can affect cognitive functions in later life.

The main limitation of the present study is that there was a duration of several days between BP measurements (just before delivery) and the day when an MRI scan was performed (several days after delivery). Further, this duration varied among the participants and BP generally drops to the normal range soon after delivery [54]. However, our results suggested that the increase in BP during pregnancy affects the brain FC and that the magnitude of change is related to BP before delivery. This is because our analysis included the MRI data acquisition date after delivery as a nuisance covariate. Although the brain FC change seems to persist for at least one week after delivery, it is currently unknown how soon the FC changes with decrease in BP and whether the change can be normalized after delivery. This important aspect, which is linked to planning of next pregnancy, should be investigated in a future study. Another limitation is that the present study does not indicate a causal relationship between dBP and brain FC change. It is possible that a low rGC value in the orbitofrontal area is a risk factor for increases in BP during pregnancy. This possibility could be evaluated by a comparison of rGC values before pregnancy and after delivery.

There is a concern that some antihypertensive drugs may affect cerebral blood flow. Recent studies [55, 56] have shown that beta blockers are associated with an alteration in functional connectivity in patients with psychiatric disease. However, because only one patient with PE was prescribed a beta blocker (labetalol hydrochloride, see **S1 Table**), the possible effect of a beta-blocker must be minor. Furthermore, all of the participants were free from any antihypertensive drugs on the day of MRI data acquisition.

Overall, the present study demonstrated a negative correlation between rGC in the orbitofrontal area and dBP. This finding suggests that perinatal BP elevation, even in the absence of other symptoms, has some effects on brain FC.

## Supporting information

S1 Table Demographic and functional connectivity related data for each of the participants. (DOCX)
