# Supplementary material for: Effects of perinatal blood pressure on maternal brain functional connectivity
Source: PLoS One. 2018 Aug 28;13(8):e0203067. doi: 10.1371/journal.pone.0203067 (PMC6112678; doi:10.1371/journal.pone.0203067)
Supplement: S1 Table — (DOCX) [file pone.0203067.s001.docx]

Supplementary Table 1. Demographic and functional connectivity related data for each of the participants

| Subject No. | Age | Blood Pressure (mmHg), systolic/diastolic (mean) | Medication | Diagnosis | rGC  on ROI A | rGC  on ROI B | FC  seeding at  ROI A rtACG | FC  seeding at  ROIA rtMFG | Variance of  global signal | Variance of  white matter |
| --- | --- | --- | --- | --- | --- | --- | --- | --- | --- | --- |
|  |  |  |  |  |  |  |  |  |  |  |
| 1 | 42 | 185/91 (122) | M and C | severe PE | 0.22561981 | 0.068832278 | 1.4369371 | 0.38915724 | 0.209570623 | 0.172066991 |
| 2 | 35 | 189/108 (135) | H, M and C | severe PE | 0.25906074 | 0.087606043 | 1.4825842 | 0.33952492 | 0.864859866 | 0.316692789 |
| 3 | 21 | 135/74 (94) |  | Normal | 0.24848922 | 0.07655371 | 2.0883925 | 0.30259866 | 0.8914165 | 0.215992617 |
| 4 | 34 | 164/101 (122) |  | PE | 0.27539417 | 0.073578455 | 1.9188287 | 0.3645325 | 0.620585282 | 0.184847237 |
| 5 | 33 | 187/115 (139) | H and C | severe PE | 0.20843497 | 0.082173631 | 1.2944793 | 0.1621118 | 0.422961037 | 0.127862272 |
| 6 | 28 | 152/102 (118) |  | GH | 0.2282505 | 0.085454784 | 1.3437914 | 0.34202293 | 0.62904188 | 0.184158855 |
| 7 | 34 | 158/92 (114) |  | PE | 0.2222486 | 0.081143245 | 1.0457915 | 0.20787637 | 0.40154095 | 0.105523346 |
| 8 | 36 | 115/68 (83) |  | Normal | 0.26290408 | 0.089175373 | 1.8667682 | 0.39634129 | 1.093678366 | 0.346259092 |
| 9 | 29 | 168/110 (129) | H, M and C | severe PE | 0.26194629 | 0.10125184 | 1.8456659 | 0.3338753 | 0.570818419 | 0.149925015 |
| 10 | 30 | 155/89 (111) | H | PE | 0.25930315 | 0.091390036 | 1.7162105 | 0.21387678 | 0.271488964 | 0.167470604 |
| 11 | 41 | 106/62 (76) |  | Normal | 0.27860138 | 0.090141878 | 1.6445854 | 0.44620001 | 0.382357609 | 0.4343074 |
| 12 | 25 | 177/112 (133) | H | severe PE | 0.21365511 | 0.080946207 | 1.4638288 | 0.17469075 | 0.709516175 | 0.315832527 |
| 13 | 41 | 147/88 (107) |  | GH | 0.27670795 | 0.081571497 | 1.9119407 | 0.4975352 | 0.71804864 | 0.227497773 |
| 14 | 36 | 187/116 (139) | H | severe PE | 0.26035199 | 0.075732373 | 0.93009299 | 0.14834769 | 0.150371458 | 0.094898931 |
| 15 | 32 | 93/53 (66) |  | Normal | 0.31900713 | 0.11508958 | 2.1512787 | 0.4919208 | 1.000904301 | 0.636740718 |
| 16 | 35 | 119/78 (91) |  | Normal | 0.24684119 | 0.099882618 | 1.7816995 | 0.36510476 | 0.626466801 | 0.288267527 |
| 17 | 33 | 188/110 (136) | H and C | severe PE | 0.24986048 | 0.069524117 | 1.2231491 | 0.38768223 | 0.208242924 | 0.11807978 |
| 18 | 31 | 174/106 (128) | C | severe PE | 0.27194113 | 0.072249897 | 1.5233945 | 0.36767873 | 0.554894452 | 0.24687874 |
| 19 | 30 | 174/117 (136) | H and C | severe PE | 0.2483699 | 0.076743066 | 1.1218975 | 0.29712453 | 0.433998859 | 0.308143902 |
| 20 | 32 | 114/83 (93) |  | Normal | 0.23608716 | 0.077096649 | 1.6299069 | 0.59541243 | 0.198278493 | 0.104875353 |
| 21 | 29 | 101/76 (84) |  | Normal | 0.31656182 | 0.081895664 | 1.5994865 | 0.62401789 | 0.636577801 | 0.444681895 |
| 22 | 33 | 110/58 (75) |  | Normal | 0.29509267 | 0.10323044 | 1.9690419 | 0.58984828 | 0.756118854 | 0.349178484 |
| 23 | 26 | 188/108 (134) |  | GH | 0.21807104 | 0.076799095 | 1.3069412 | 0.27805737 | 0.471942416 | 0.202833976 |
| 24 | 29 | 163/89 (113) | H and L | severe PE | 0.26111698 | 0.10346134 | 1.635164 | 0.57539225 | 1.089934449 | 0.199822601 |
| 25 | 41 | 177/108 (131) | H, M and C | severe PE | 0.21274288 | 0.069044933 | 1.2674018 | 0.25622612 | 0.353607607 | 0.166615446 |
| 26 | 27 | 198/118 (144) | M and C | severe PE | 0.21269919 | 0.072063975 | 1.1164063 | 0.1978253 | 0.392863386 | 0.152091725 |
| 27 | 32 | 171/117 (135) | C | severe PE | 0.22565386 | 0.075753108 | 1.2896255 | 0.27036732 | 0.393992671 | 0.721149505 |
| 28 | 21 | 164/80 (108) | C | severe PE | 0.2944347 | 0.087507993 | 2.9367654 | 0.54698288 | 0.773477886 | 0.780103432 |
| 29 | 38 | 148/95 (112) |  | GH | 0.27779192 | 0.091345482 | 1.7801987 | 0.34767011 | 1.275299741 | 0.688498227 |
| 30 | 39 | 198/111 (140) | H, M and C | severe PE | 0.22865959 | 0.090014875 | 1.6396488 | 0.23433101 | 0.536589373 | 0.255695747 |
| 31 | 29 | 173/107 (129) |  | severe PE | 0.23721406 | 0.072052628 | 1.4997128 | 0.2208932 | 0.377529476 | 0.102700143 |
| 32 | 30 | 121/70 (87) |  | Normal | 0.26925683 | 0.094879143 | 1.8934788 | 0.5142538 | 0.489290016 | 0.184208686 |
| 33 | 30 | 107/67 (80) |  | Normal | 0.25889704 | 0.1027342 | 1.7957382 | 0.55290222 | 0.95613712 | 0.233391639 |
| 34 | 30 | 116/90 (98) |  | Normal | 0.21451427 | 0.078473508 | 1.3787178 | 0.11496416 | 0.970427582 | 0.471549311 |
| 35 | 30 | 98/67 (77) |  | Normal | 0.29263437 | 0.094548672 | 1.7816861 | 0.44590598 | 0.568529864 | 0.167170844 |
| 36 | 31 | 165/76 (105) |  | PE | 0.28457612 | 0.091143727 | 2.3608577 | 0.53105903 | 0.491135724 | 0.159777283 |
| 37 | 35 | 203/102 (135) | H and M | severe PE | 0.20254235 | 0.069776885 | 1.550521 | 0.38489446 | 0.661204355 | 0.433753637 |

ACC = anterior cingulate gyrus, C = dihydropyridine, H = hydralazine hydrochloride, L = labetalol hydrochloride, M = methyldopa, MFG = middle frontal gyrus
